# Salvage surgery for continent ileostomies (CI) after a first successful revision: more long-term blame on the reservoir than the nipple valve

**DOI:** 10.1007/s00384-021-04054-x

**Published:** 2021-10-30

**Authors:** Karl-Wilhelm Ecker, Mathias Tönsmann, Nils Karl Josef Ecker, Gabriela Möslein

**Affiliations:** 1 University of Saarland, Homburg, Saar, Germany; 2 Surgical Dept, MediClin Müritz-Klinikum, Weinbergstraße 19, 17192 Waren, Germany; 3 Hagen, Germany; 4 Ahrensburg, Germany; 5 Center for Hereditary Tumors, Ev. Krankenhaus BETHESDA, University of Düsseldorf, Heerstraße 219, D-47053 Duisburg, Germany

**Keywords:** Continent ileostomy (CI), Kock Pouch, CI survival, CI revisional surgery, CI and underlying disease

## Abstract

**Purpose:**

The aim of the study was to investigate the underlying cause of long-term complications in patients requiring at least one revision surgery of a continent ileostomy (CI) and to analyze functional outcome.

**Methods:**

Only patients with CI at least one revision were included in the retrospective data analysis. Four different classes of complications (Cl A–D) were defined: Cl A = Nipple valve (NV), Cl B = pouch, Cl C = outlet (stoma), and Cl D = afferent loop (AL). Associations between underlying disease and origin of complications were analyzed. Cumulative probabilities were calculated using Kaplan–Meier analysis.

**Results:**

A total of 77 patients were identified with a follow-up of 30 years, requiring 133 surgeries for 148 complications (c.). Cl A 49 c. (33.1%), Cl B 50 c. (33.8%), Cl C 39 c. (26.4%), and Cl D 10 c. (6.8%). Cl A and C complications were not correlated to underlying disease, whereas Cl B and D complications were only found in ulcerative colitis (UC) and Crohn’s disease (CD). The cumulative probability of a second revision showed a linear rise, reaching 62.5% after 20 years. Cl A and B complications both reached 42.1%. Eleven (14.3%) patients (10 Cl B) had pouch failure in a follow-up period of 11.5 ± 8.7 years (1–31 years), whereas 66 (85.7%) had successful revisional surgery. Overall CI survival was 78.8% at 44 years.

**Conclusion:**

CI survival is limited by inflammatory complications of the pouch based on the underlying disease and not by mechanical limitations of the NV.

**Trial registration numbers:**

None.

## Introduction

Continent ileostomy (CI), introduced by Nils Kock more than half a century ago, once aimed to radically change the concept of defecation following proctocolectomy. CI was implemented as the primary procedure and alternative to the standard incontinent end ileostomy. However, with the original technique, a high rate of incontinence persisted, which is why the initiator of the concept of a reservoir (pouch) construction added the intussusception of the outlet canal (nipple valve NV) in a second evolutionary step [1]. Thus, the procedure referred to today as “Kock Pouch” (KP) was born. Unfortunately, NV sequelae turned out to be the “Achilles’ heel” of the operation [2]. Despite numerous technical improvements, the persistent and predominant complication of “slippage” is to date the most frequent indication for revisional surgery [3]. Some years later, ileo-pouch-anal anastomosis (IPAA) became the preferred procedure in the event of proctocolectomy due to the preservation of the natural defecation pathway and CI became increasingly less important [4]. However, not all patients may benefit equally from the advantages of sphincter preservation, so that there still are sensible indications in selected patients for fashioning a CI, both as a salvage procedure after IPAA and as a primary procedure for any cause requiring proctocolectomy and a permanent ileostomy. [5].

The authors were attracted by the concept of both IPAA and CI and implemented these procedures at their institution [6]. The first author spent several weeks as a visiting clinician in Gothenburg under the supervision of Prof. Nils Kock. From the very beginning, the focus was laid on improving the outcome by perfectioning the technique and simultaneously optimizing complication management. Based on this collaboration and the “handing-over” of CI patients from Nils Kock to K.-W. Ecker, the authors now report on the unique long-term outcome of CI patients with revisional surgeries of a maximum of 44 years. The prospectively maintained patient cohort constitutes the rare opportunity to report on the broad spectrum of Indications and procedures for operative revisions and their impact on long-term prognosis. The specific question investigated was if the nipple valve (NV) truly is the limiting factor of survival of CI?

## Patients and methods

### Study design and patients

This retrospective study includes all patients with a minimum of one CI revision performed by one of the authors (KWE) between 1986 and 2015 with a follow-up until 2020**.** All patients with primary CI construction at both the own and external institutions were included in the series. The study takes into account only for patients requiring revisional surgery at least > 30 days after the primary procedure, excluding the early postoperative complication group. We defined two groups: delayed surgery between 30 days and 12 months and long-term > 12 months p.o.

### Classification of complications

Complications were classified into four categories.

- Class A: Complications of Nipple valve (NV)
- Class B: Complications of the pouch
- Class C. Complications of the stoma
- Class D: Complications of the afferent loop

### Data collection and statistics

Data from patient records were entered into a database in SPSS (statistics program of IBM™). In addition to descriptive statistics, cumulative probability rates were determined using a Kaplan–Meier analysis.

## Results

### Patients and modalities of previous operations

In total, 77 CI patients with a mean age of 46.4 ± 11.7 years were included, with their primary surgery performed 8.7 ± 11.2 years previously. Of these 48 were performed at our institution, 22 were patients of Prof Kock in Gothenburg and 7 were patients from other German surgeons. A total of 64 patients had inflammatory bowel disease (IBD): 48 ulcerative colitis (UC) and 16 Crohn’s colitis (CC). Thirteen patients suffered from non-inflammatory bowel disease (Non-IBD), of which 11 had familial adenomatous polyposis (FAP), one slow transit constipation (STC) and another rectal cancer (RC). A total of 22 patients underwent surgical revision for delayed postoperative complications and 55 for long-term complications. CI patients originating from Prof Kock were exclusively revised for long-term complications; all other patients had required surgery both due to delayed postoperative and long-term complications (Table 1).

**Table 1:** Patients and surgical history

	***n***	**(%)**
**Patients**	**77**	**(100.0)**
Male	35	(45.5)
Female	42	(54.5)
**Underlying diseases**		
Ulcerative colitis	48	(62.3)
Crohn’s colitis	16	(20.8)
Non-IBD Fam. polyposis Rectal cancer Slow transit constipation	13 11 1 1	(16.9)
**Previous operation**		
Primary CI-construction	62	(80.5)
Conversion of IPAA	15	(19.5)
**Previous surgeon**		
KW Ecker	48	(62.3)
NG Kock	22	(28.6)
Various other surgeons	7	(9.1)
**Time allocation of the complication**		
Delayed postoperative	22	(28.6)
Long-term	55	(71.4)
**Age and time information (years)**	**(M ± SD)**	**Median (range)**
Age at time of first revision	46.4 ± 11.7	48 (20–71)
Interval CI constr. to first reoperation	8.7 ± 11.2	4 (0.5–37.1)

Among the 77 patients, 133 revision surgeries (1.7/pat) were performed in up to five successive procedures, which are listed in Table 2. Both the mean and the median age of the patients increased by approximately 20 years from the first to the fifth revisional surgery. It was interesting to note that both the standard deviation and the range of age decreased in the sequence of the revision procedures. Surprisingly, patients of approximately 70 years of age were overrepresented throughout the sequence of reoperations as a clue that older patients may become more susceptible to complications after a long uneventful interval.

**Table 2:** Cumulative 133 revision operations in correlation to patient age

Sequence of reoperations *n* = 133	*n* (%)	Age of patients (years)	
		**Mean ± SD**	**Median (range)**
First	77 (100.0)	46.4 ± 11.7	48.0 (20–71)
Second	31 (40.3)	48.3 ± 12.1	49.5 (26–74)
Third	15 (19.5)	53.5 ± 10.7	57.0 (33–69)
Forth	8 (10.4)	56.4 ± 8.7	59.0 (44–70)
Fifth	2 (2.6)	67.0 ± 8.5	67.0 (61–73)

### Categories of complications and choice of procedure for revisional surgery

In total, 133 revision surgeries were performed for 148 different complications, all of which were individually corrected with the indicated procedures (Table 3).

**Table 3:** Site (localization) of 148 complications and associated procedures

Site of special complication (revisions 1–5)	*n* (%)	Type of special procedure	*n*
**Class A: Nipple valve** Intubation problem Nipple slippage Valve prolapse other (ulceration, stenosis)	**49 (33.1)** 19 19 8 3	Valve restabilization New valve construction	22 27
**Class B: Pouch**	**50 (33.8)**		
Fistula	20	Fistula excision and closure	12
Surrounding valve base		New valve construction	7
		Pouch excision and IS	1
Fistula Pouch-cutaneous/enteric	15	Fistula excision and closure	11
		Pouch excision and new CI	2
		Pouch excision and IS	2
Pouchitis	4	Pouch excision and IS	4
Pouch detachment from abdominal wall	3	Refixation of pouch	3
			4
Other	8	Various repairs Pouch excision and IS	4
**Class C: Outlet/Stoma**	**39 (26.4)**		
Stenosis, retraction	26	Plastic reconstruction	26
Hernia	8	Hernia repair	8
Fistula/abscess	5	Fistula repair	3
		Plastic reconstruction	2
**Class D: Afferent loop**	**10 (6.8)**		
Stenosis not CD related	1	Resection	1
Recurrence of CD (stenosis and/or fistula)	9	Resection (S-pouch) Bypass (K-pouch)	6 3
**All sites**	**148 (100.0)**		**148**

In the total amount of 148 complications, requiring revision surgery class A complications (*n* = 49) accounted for one third (33.1%). All off these were attributed to impaired valve stability (intubation problem, slippage, prolapse). Valve-preserving techniques always had priority over valve reconstruction. A total of 22 of 49 (44.9%) NV instabilities were successfully treated by simple re-stabilization. Only if this straightforward repair was not feasible, or failed (*n* = 27; 55.1%), construction of a new NV was required.

Complications of the pouch (class B-complications) accounted with 50 cases (33.8%), corresponding to another approximate one third of indications. Penetrating complications such as fistulas were predominant (70.0%), occurring in 35 cases. Of these, 20 bypassed the NV at the base and produced incontinence, while 15 were pouch-cutaneous or pouch-enteric fistulas. Four patients suffered from refractory disabling pouchitis (5.2% of total cohort), 3 had problems due to detachment of the pouch from the abdominal wall, and 8 suffered from various other pouch-related problems. Preservation of the CI, wherever reasonable, was given preference to more invasive reconstructive techniques. This included construction of a new NV in 7 of 20 cases with fistulae at the base of the valve in occasions of unfeasibility of plastic fistula closure. A complete CI reconstruction was required 2 cases, in which pouch-cutaneous or -enteric fistulas failed plastic closure (*n* = 11). However, over time, 11 out of 77 patients (14.3%) lost their pouch, mostly due to severe therapy-refractory inflammatory complications of IBD. One patient with STC who suffered from considerable abdominal distension opted for abandoning the KP. In these cases, the entire reservoir and nipple valve were resected and an IS was the definitive solution.

Stoma complications (class C) accounted for a quarter of all complications (39, *p* = 26.4%) and were easily corrected by established techniques with plastic reconstructive components. Only 10 cases were attributed to problems related to the afferent loop (class D-complications) and as such represented the smallest patient group (6.8%). All of these latter patients had the underlying diagnosis of CD and as such should be treated and interpreted as a unique subgroup of patients [7].

### Peri-operative morbidity

As expected, the 26 local stoma revisions did not lead to any significant intraoperative complications with five minor and easily correctable postoperative incidents (stenosis, retraction). Among the 107 abdominal revisions, 16 intraoperative complications (15.0%) were reported, specifically adhesion-related injuries of major blood vessels, the intestine, and ureters. All iatrogenic complications were successfully resolved simultaneously during the procedure. Minor postoperative complications occurred in 16 patients (15.0%), including wound and urinary infections. They were successfully treated with standard procedures of care. Major postoperative complications requiring relaparotomy were recorded in 9 patients (8.4%). Five anastomotic leaks required immediate revision and were successfully treated by abdominal lavage and anastomotic repair. Other singular rare complications included nipple valve necrosis, hernia formation, and pouch occlusion pre-perforation. The latter were successfully treated surgically in a semi-elective manner and did not lead to any mortality (Table 4).

**Table 4:** Perioperative morbidity in 107 abdominal revision operations

	***n***	**(%)**
**All abdominal revision surgeries**	**107**	**(100.0)**
**Intraoperative complications** Vascular lesions/bleeding Uretero-vesical lesions Intestinal lesions Failed valve reconstruction	**16** 9 3 3 1	**(15.0)**
**Postoperative minor complications**	**16**	**(15.0)**
Surgical site infection	11	
Limited bleeding	2	
Urinary tract infection	3	
**Postoperative major complications**	**9**	**(8.4)**
Anastomotic break down	5	
Nipple valve necrosis	1	
Imminent pouch perforation	1	
Hernia peristomal/abdominal	2	

### Long-term course and survival of CI following first revisional surgery

**Fig. 1:**
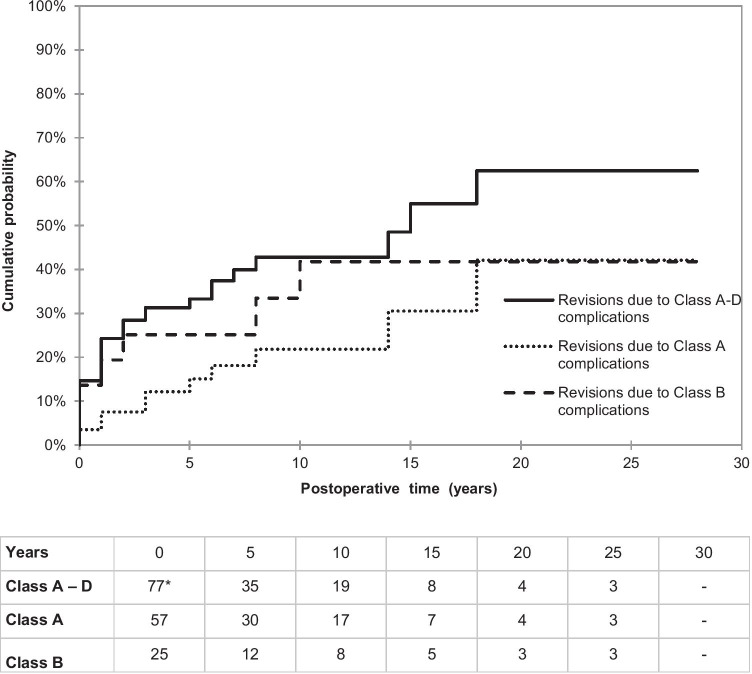
Cumulative probability of the second revision surgery based on the complication categories A and B and all (A–D). Asterisk represents 3 patients with simultaneous class A and B complications as second indication for revision

Ten patients were lost to follow-up for unknown reasons, but with well-functioning CI at last follow-up. Taking this into account, the cumulative probability for a second revisional surgery of any complication (class A-D) increased globally to 31.4% in the first 5 years and then continued to increase at a slower pace 42.8% and 62.5% by the 15th and 20th years, respectively. In contrast, the nipple valve (class A) complications developed significantly later, reaching 21.8% after 10 years and 42.1% after 20 years. This includes only valve instabilities. The probability of pouch revisions (class B) was more frequent in the first years than valve revisions (class A), reaching a concordant maximum of 42.1% after 10 years (Fig. 1).

**Fig. 2:**
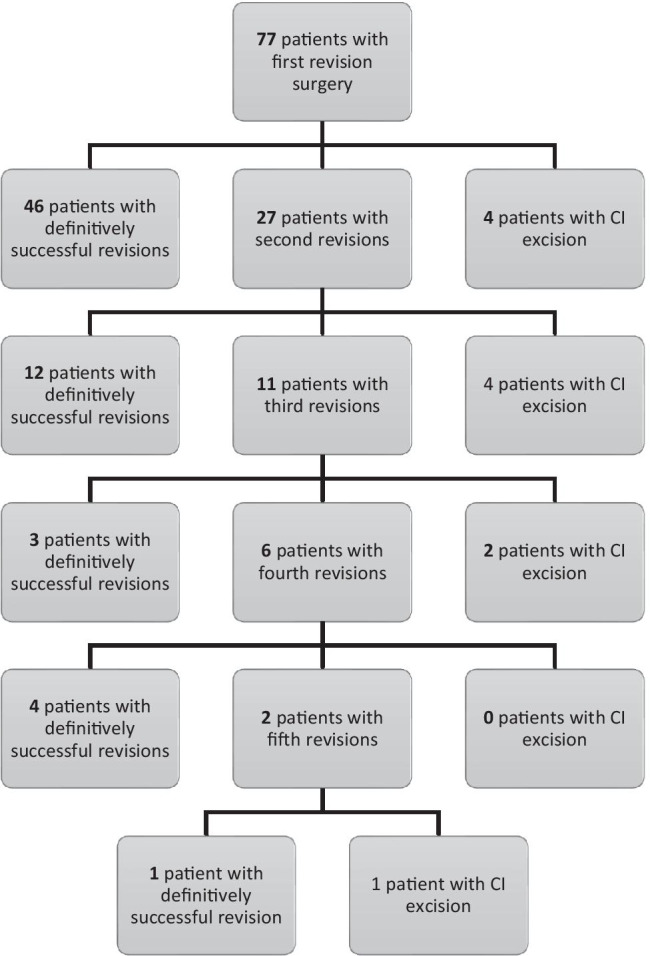
Flowchart of patients and pouch outcome

Follow-up after first revisional surgery was 11.5 ± 8.7 years (1–31 years). During this time, 31 patients (40.3%) underwent a second revisional surgery (*n* = 27) or CI-excision (*n* = 4). Figure 2 illustrates the chronological order of all sequential surgeries based on success (functional repair) and overall failure (CI excision). At the end of the observation period, 85.7% (*n* = 66/77) patients had regained functionality of CI leading to a crude pouch failure rate of 14.3% (*n* = 11/77).

**Fig. 3:**
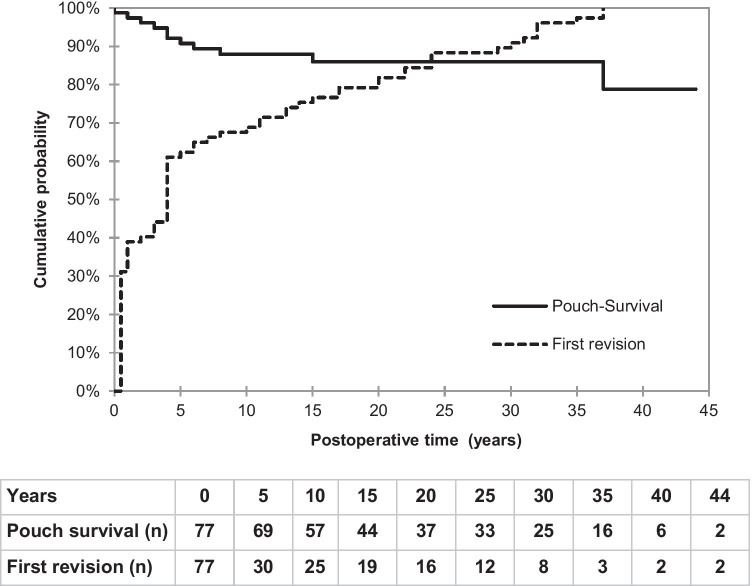
Cumulative probability of the first revision surgery after construction of the CI in correlation to overall CI survival probability

In the following life table analysis, the probability of revisional surgery (starting point being the time point of the primary CI construction) was calculated additionally to the cumulative CI survival rate. Since the inclusion criteria for this study is limited to patients who required at least one first surgical revision, the curve reaches the 100% mark. However, it is of important note that CI patients are most frequently affected in the first year after initial construction and reach 40% after 5 years already. Subsequently, the curve flattens considerably until reaching the pre-defined 100% after 37 years. Correspondingly, the pouch failure rate was highest in the first 5 years (9.2%), translating to a cumulative survival rate of CI of 90.8%. Due to successful revisional surgery, the survival probability only further decreased to 78.8% after 44 years of follow-up (Fig. 3).

## Discussion

CI or KP has been abandoned as primary reconstructive procedure ever since the implementation of IPAA as the preferred reconstructive procedure for proctocolectomy [5, 7, 8]. An important argument for the change in strategy was that IPAA preserves the natural defecation route, thereby preventing the requirement of a permanent stoma. Also, the high revision rates of CI were arguments raised against broader implementation and acceptance. [4, 9]. Nils Kock himself and other early advocates such as R. Dozois and V. Fazio [10–12] recognized these sequelae as followed by other surgical opinion-leaders further on [13–17]. Revision rates between 21 and 70% and pouch loss rates between 5 and 20% were reported [9]. These authors almost unanimously agreed that selected patients benefit largely from excellent and undisturbed function for many years or even decades, whereas others require revision surgery, even repetitively. Also, there seems to be agreement on the NV as the underlying cause for most functional complications and therefore is regarded the “Achilles’ heel” of the operation and major underlying cause of CI failure [2, 3, 18]. In the authors view, not enough attention has been paid to the pouch itself and the influence of the underlying diseases. Thus, the discussion is mostly triggered by technical considerations only, albeit CD has largely been considered an unspecified risk factor for all complications and pouch failures, but based on limited and biased data [19–21]. The data presented here focusses exclusively on the analysis of all patients requiring revisions and is a unique large data set with (very) long follow-up.

### Classification of complications

The classification of complications into four anatomically defined categories is the first report allowing correlation of both anatomical resp. technical issues and underlying disease.

1. Complications of NV (class A) surprisingly do not dominate the complication spectrum as may be assumed from the historical literature [11, 15, 16]. The comparatively low crude rate of 33.1% in the present study results on one hand from the exclusion of fistulas surrounding the NV. Additionally, it is of note that the fistula-enhancing Marlex meshes were no longer primarily implanted for increased stabilization [11]. In our view, the fistulas surrounding the NV must be correctly attributed anatomically to pouch fistulas, since the NV itself remains completely intact. On the other hand, by extending the observation period in this study to present day, improved stapling techniques also have an effect on reducing the rate of NV instabilities. The clinical observation that the underlying disease, especially CD, has no influence on the stability of NV must be emphasized and prospectively be taken into account.
2. Complications of the pouch (class B) are mainly mentioned in the literature as pouchitis in UC and therefore only play a minor role in the management of surgical complications [22]. In the present study, however, the crude rate of pouch complications of 33.8% requiring surgical treatment quantitatively reaches the magnitude of NV complications. This is due to the inclusion of all types of fistulas originating from the pouch wall as well as to the comparatively high amount of contribution of patients with CD. Moreover, qualitatively, they are the only ones to harbor a significant potential for pouch failure. The underlying diseases, mainly IBD, are to be taken into account for this outcome. Despite the remaining diagnostic uncertainty, no significant difference can be attributed to UC versus CD for refractory pouchitis or unsuccessful management of pouch fistulae and subsequent sacrifice of the pouch. Only rarely therefore does construction of a new CI seem justifiable for this scenario.
3. Complications of the stoma (class C) represent approximately 25% of the complications, often associated with others. These may virtually always be successfully dealt with local surgery and correspond to complications of a conventional ileostomy [23–25].
4. Complications of the afferent loop (class D) are rare, and systematic reference is not found in the comparative literature. They are associated with IBD. In contrast to class B complications, they should be considered pathognomonic for CD. If not combined with irreparable class B complications, these are successfully managed surgically. Apparently, they have a similar pathogenesis as pre-anastomotic recurrence of neo-terminal ileum after ileocecal resection [26].

### Revision surgery and associated morbidity

From a surgical-technical point of view, the complications of the nipple valve (class A) in particular require a more in-depth discussion. As early as 1976, Kock himself described the mechanisms of development of instabilities of the nipple valve including fistula formations. He indicated basic techniques for surgical correction [12]. With the aim of preventing desusception of the NV, Thompson et al. secondarily added Barnett's collar formation as an alternative procedure in 1992 [27, 28] and Fazio et al. described wall fixation of the nipple valve using a stapler in the same year [29]. The authors of the present study agree with the view expressed in the literature that valve-preserving correction should always take precedence over valve remodeling in the surgical revision of instabilities. For new NV constructions, Denoya et al. have used the term of a “turnaround procedure” in cases where the valve is constructed from the afferent loop and “pedicle repair” in cases where it is constructed from a transposed small bowel segment [3].

Complications of the pouch, stoma, and afferent loop (classes B-D) are successfully managed surgically by following the general principles of visceral and stoma surgery. In this context, the pouch fistulas surrounding the NV represent a technical challenge. The layer between the pouch shoulder and the outlet must be carefully dissected in order to excise and suture the fistula outflow and inflow separately. Only if this technique fails, may it become necessary to construct a new NV as in the case of remaining instability. In the authors’ experience, resection of the afferent loop is much more successful and feasible with an underlying S design of the pouch and as such a strong argument for the technical preference adopted, since corrections in this setting do not require the trauma of mobilizing the entire pouch. This is a clear advantage over the original K-pouch (in addition to other benefits), which has lead the authors to implement the S-pouch as the standard pouch [5].

Differentiated data on the perioperative morbidity of different revisional procedures are not retrievable in dedicated literature, since reports combine standard primary constructions and revision procedures. Also, no distinction is made between complications that can be treated conservatively and those that require revision. An example of this is the rate of postoperative major complications of 19% from the Cleveland Clinic/USA [11]. Only in the study from Mount Sinai Hospital in New York, 31 revision surgeries were reported with one intraoperative and three postoperative major complications (corresponding to 3.2% and 9.7%) [3]. In the present study, these favorable results are confirmed in a cohort more than three times larger with 107 revision operations. Potentially, the lower morbidity of revision surgery compared to primary CI construction is due to the fact that the surgery is performed on a long healed new organ.

### Importance of revision surgery for the fate of CI

CI revisional surgery must rest in the hands of the most experienced specialized pouch surgeons after dedicated training and mentoring in order to be successful. Critical experienced patient assessment and selection in addition to dedicated communication with the patient (expectations) combined with perfect surgical technique and meticulous postoperative management are mandatory. [30]. To our knowledge, the paramount challenge of revision surgery has been acknowledged by only one paper from Mount Sinai Hospital in New York. Denoya et al. report on 31 patients who required (re-)revision surgery after a complication-free course of at least ten (11.7–28.2) years after CI creation (*n* = 21) or the last revision (*n* = 10) [3]. However, the authors focus was explicitly dedicated to the need for revision of the nipple valve. In this setting, 19 patients (61.3%) were restored by a single abdominal revision surgery, and 12 patients required additional revisions. Two patients (6.5%) required pouch excision and end ileostomy, corresponding to a long-term CI survival of 93.5%.

In the present study, cumulative probabilities for second revision surgery and pouch survival are analyzed based on the entry criteria of at least one CI revision. As such, the cohort analyzed may in itself be a “negative selection.” This must be kept in mind, since all CI pouches never requiring revisions are a priori excluded and the outcome here reported compares to unselected overall series in literature [13, 15, 16, 31]. Unexpectedly, future pouch complications (class B) occurred more rapidly than further nipple valve complications (class A) leading to a second revisional surgery. The reason for this interesting observation may be that valve complications had apparently been surgically resolved, whereas pouch complications were fatefully linked to the underlying condition (UC and/or CD). Potentially, however, successful conservative management of these problems hopefully may be expected based on more targeted medication. [32]. According to literature, approximately every second patient (every third in this study) after primary CI construction must expect a revision operation of the nipple valve within 20 years [16, 31]. This then sets the clock back to zero. In this study, it could be shown that after revision surgery, the clock for complications of both the nipple valve (class A) and the pouch (class B) continues to run, but with a lower probability for complications in the following 20 years.

Based on these correlations, pouch sacrifice is to be attributed to the underlying disease IBD (UC and CD alike) and not to the challenging design and revision technique of the nipple valve. As shown by the “revision history” (Fig. 2) over a maximum of 44 years, valve revisions are in principle repeatedly and successfully feasible; however, pouch loss due to pouch failure may occur at any time. It must be conceded that the relationship between success and failure of revision surgery worsens with an increasing number of revisions (due to the association with IBD). Nevertheless, the “pouch survival” of CI revised at least once, calculated according to the Kaplan–Meier Methods, only decreases to the 80% range even with a follow-up of multiple decades. Thus, in this study, the long-lasting durability of CI, as described in the literature for patient series after primary CI construction [13, 15, 16, 31], could be confirmed even in the case of the “negative selection” inclusion criteria.

### Strengths and weaknesses of the study

The strength of the study results from the unique feature of analyzing the largest patient series reported to date with CI requiring revisional surgery. The variations of complications encountered have been classified and systematically addressed, allowing for better comparability of future reports. The retrospective design may be a weakness, albeit an unavoidable one. However, the very long follow-up allows for unanticipated attribution of disease rather than technical issues as a decisive factor for pouch failure in CI patients.

## Conclusions

Against the background of recognized frequent need for revision of CI, the presented study proves that procedural complications of NV need by no means be the limiting factor in the long-term course of patients. Almost all cases of instability of the NV were successfully treated surgically, whereas complications of the reservoir and overall failure were correlated to the underlying disease. The authors conclude that different than the overall perception, the NV is not the unsurpassable disadvantage of the procedure. Successful revision surgery in the hand of the experienced, however, is an integral part of the overall surgical concept of CI.

## Data Availability

The data and all materials used are secured digitally by the second author (Mathias Tönsmann).

## Code availability

No code available.
